# Vascular Surgery in Japan: 2015 Annual Report by the Japanese Society for Vascular Surgery

**DOI:** 10.3400/avd.ar.21-00077

**Published:** 2021-09-25

**Authors:** The Japanese Society for Vascular Surgery Database Management Committee Member, NCD Vascular Surgery Data Analysis Team

**Keywords:** peripheral arterial disease, stent graft, endovascular treatment, aneurysm, varicose vein treatment

## Abstract

**Objectives:** This is an annual report indicating the number and early clinical results of annual vascular treatment performed by vascular surgeon in Japan in 2015, as analyzed by database management committee (DBC) members of the JSVS.

**Materials and Methods:** To survey the current status of vascular treatments performed by vascular surgeons in Japan, the DBC members of the JSVS analyzed the vascular treatment data provided by the National Clinical Database (NCD), including the number of treatments and early results such as operative and hospital mortality.

**Results**: In total 124,299 vascular treatments were registered by 1,038 institutions in 2015. This database is composed of 7 fields including treatment of aneurysms, chronic arterial occlusive disease, acute arterial occlusive disease, vascular injury, complication of previous vascular reconstruction, venous diseases, and other vascular treatments. The number of vascular treatments in each field was 22,041, 15,671, 4,779, 2,313, 857, 48,837, and 29,801, respectively. In the field of aneurysm treatment, 18,907 cases of abdominal aortic aneurysm (AAA) including common iliac aneurysm were registered, and 57.6% were treated by endovascular aneurysm repair (EVAR). Among AAA cases, 1,850 (9.8%) cases were registered as ruptured AAA. The operative mortality of ruptured and un-ruptured AAA was 16.0%, and 0.6%, respectively. 33.6% of ruptured AAA were treated by EVAR, and the EVAR ratio was gradually increasing, but the operative mortality of open repair and EVAR for ruptured AAA was 16.6%, and 14.5%, respectively. Regarding chronic arterial occlusive disease, open repair was performed in 8,230 cases, including 1,194 distal bypasses to the crural or pedal artery, whereas endovascular treatment (EVT) was performed in 7,441 cases. The EVT ratio was gradually increased at 47.4%. Venous treatment including 47,046 cases with varicose vein treatments and 531 cases with lower limb deep vein thrombosis were registered. Regarding other vascular operations, 29,801 cases of vascular access operations and 1,511 lower limb amputation surgeries were included.

**Conclusions:** The number of vascular treatments increased since 2011, and the proportion of endovascular procedures increased in almost all field of vascular diseases, especially EVAR for AAA, EVT for chronic arterial occlusive disease, and endovenous laser ablation (EVLA) for varicose veins. (This is a translation of Jpn J Vasc Surg 2020; 29: 161–179.)

## Introduction

The National Clinical Database (NCD; a general incorporated association) was inaugurated in 2011 and initiated the registration of surgical cases. In response, the Japanese Society for Vascular Surgery (JSVS) started tallying NCD-registered cases of vascular surgery and presenting annual reports on vascular surgery at academic conferences.^1–7)^ This paper documents the results of tallying and analysis, which was conducted by the members of the JSVS Database Management Committee, of the NCD-registered cases of vascular surgery performed from January to December 2015.

## Methods

From the NCD-registered surgical cases in 2015, data on the cases of vascular surgery were extracted with the commission of the JSVS (an NCD-affiliated organization). On the basis of these data, the cases were classified into the following seven categories, and the tallied results were checked and analyzed by the members of the JSVS Database Management Committee. The categories were as follows: 1) revascularization for arterial aneurysm, 2) revascularization for chronic arterial occlusion, 3) revascularization for acute arterial occlusion, 4) treatment for vascular trauma, 5) surgery for revascularization-related complications, 6) venous operation, and 7) other vascular diseases and related operations.

The tallied results presented include the numbers of cases by surgical modality, causes of disease, operative death count, hospital death count, and materials used. A operative death refers to any death in which the patient died within 30 days of surgery. Regardless of the cause of death or hospitalization status, all mortalities within 30 days of surgery are included. A hospital death signifies any postoperative death during the hospitalization irrespective of the time of occurrence.

The table presented contains some discrepant values, such as the total of the causes of disease and used materials that are inconsistent with the total number of cases. These inconsistencies were thoroughly investigated by the committee and the NCD and were eventually attributed to any of the following four reasons: 1) allowing multiple choices, 2) allowing blank entries, 3) omissions or erroneous entries by the data inputter, and 4) using multiple materials for a single operation or treating multiple sites. Since 2013, measures have been taken to avoid errors, such as allocating/newly introducing options that are less prone to misunderstanding and constructing a program that prohibits blank entries from being registered as much as possible.

**Table 1** lists the items whose registration/tallying methods have been altered since 2015.

**Table:** Table 1 New items or changes in 2015 annual report

New items	Table number	Status until 2014
Revision reason	Table 3-1, 3-2, 3-3, 3-4, 3-5	Not existed
Host artery stenosis/occlusion	Table 3-1, 3-2, 3-3, 3-4, 3-5	Not existed
Graft stenosis	Table 3-1, 3-2, 3-3, 3-4, 3-5	Not existed
Graft occlusion	Table 3-1, 3-2, 3-3, 3-4, 3-5	Not existed
EVT stenosis	Table 3-1, 3-2, 3-3, 3-4, 3-5	Not existed
EVT occlusion	Table 3-1, 3-2, 3-3, 3-4, 3-5	Not existed
Poor symptom recovery	Table 3-1, 3-2, 3-3, 3-4, 3-5	Not existed
Others	Table 3-1, 3-2, 3-3, 3-4, 3-5	Not existed
Thromboendarterectomy for chronic lower limb ischemia	Table 3-4	Not existed
Other including replacement, thrombolysis and other	Table 3-4	Not existed
Debranch for TEVAR or EVAR	Table 3-6	Not existed
Ascending aorta-brachiocephalic-left common carotid (-left subclavian) arterial bypass	Table 3-6	Not existed
Right axillar-left common carotid (-left axillary) arterial bypass	Table 3-6	Not existed
Right common carotid-left common carotid (-left subclavian) arterial bypass	Table 3-6	Not existed
Left common carotid-left subclavian arterial bypass or transposition	Table 3-6	Not existed
Right axillar (subclavian)-left axillar (subclavian) arterial bypass	Table 3-6	Not existed
Abdominal aorta (iliac) (-celiac)-superior mesenteric-renal arterial bypass	Table 3-6	Not existed

## Tallying/Analysis Results

The total number of NCD-registered cases of vascular surgery was 124,299 in 2015 (9.5% increase from the previous year), exceeding 120,000 for the first time and accounting for 8.5% of the total NCD-registered surgical cases in the same year. Moreover, the number of institutions that registered the cases of vascular surgery amounted to 1,038, showing that 28.0% of the institutions registering surgical operations registered the cases of vascular surgery. Of these 1,038 institutions, 500 (48.2%) were certified training facilities for cardiovascular surgery that contributed to our data as of 2015. The analysis results are interpreted by various categories as mentioned below. For statistical analysis, a chi-squared test was used, with a p value of <0.05 regarded as statistically significant.

## Ethical Review

The NCD-registered data on vascular surgery is disclosed and analyzed on an opt-out basis. Our vascular surgery annual report was approved by the ethical review board of Kansai Medical University Hospital on April 6, 2020 (reference number: 2019276).

## 1. Treatment for Arterial Aneurysm (Table 2)

### 1) Thoracic aortic aneurysm

Most of the data on thoracic aortic aneurysm are registered in the Japan Cardiovascular Surgery Database (JCVSD), and some of the cases handled by vascular surgeons are registered in this cardiovascular surgical database via the NCD (**Table 2**). Therefore, surgical operations for thoracic aortic aneurysm performed throughout Japan are presently being registered in a fragmented manner, thus making it difficult to grasp the accurate overview of the situation. In the future, we need to consult with the JCVSD to facilitate the construction of the overview regarding the nationwide status of thoracic aortic aneurysm surgery.

**Table:** Table 2 Treatment for aneurysm Table 2-1 Aortic aneurysm

Region of aortic aneurysm	Cases	Gender		Mortality		Ruptured aneurysm			Dissection*^3）^	Etiology							
		Male	Female	30-day mortality	Hospital mortality	Cases	30-day mortality	Hospital mortality		Degenerative*^4）^			Inflammatory	Vasculitis	Infected	Connective tissue disease*^5）^	Others
										Cases	30-day mortality	Hospital mortality					
Ascending aorta*^1）^	88	52	36	5	7	11	2	3	62	76	4	5	0	0	0	0	12
Aortic arch*^1）^	458	357	101	31	40	38	9	12	155	394	24	31	0	0	8	17	39
Descending thoracic aorta*^1）^	526	371	155	18	25	60	7	9	211	436	13	19	2	1	13	24	50
Thoracoabdominal aorta*^1）^	332	254	78	24	35	51	10	12	98	279	17	28	3	1	15	11	23
Abdominal aortic aneurysm*^2）^	18,907	15,558	3,348	405	531	1,850	296	368	807	18,095	361	466	264	13	269	33	233
with renal artery reconstruction	378	317	61	13	23	40	6	9	50	348	12	21	9	0	9	1	11
with renal artery clamping	1,374	1,170	204	63	90	204	41	54	93	1,265	52	75	33	2	46	1	27

＊1) These data are not including cases recorded in JCVSD database in which most cardiac surgeons were entering their cases. ＊2) Including common iliac artery aneurysm. ＊3) Including both acute and chronic aortic dissection. ＊4) Most likely atherosclerosis. ＊5) Connective tissue abnormalities such as Marfan syndrome.

**Table:** Table 2-1 Aortic aneurysm (continued)

Region of aortic aneurysm	Treatment procedure						Graft materials*^7）^		
	Replacement			Exclusion with bypass	Stent graft	Hybrid*^6）^	Polyester	ePTFE	Others
	Cases	Y-graft	T-graft						
Ascending aorta*^1）^	2	0	0	0	11	7	55	9	6
Aortic arch*^1）^	12	0	0	2	260	149	79	66	12
Descending thoracic aorta*^1）^	26	0	0	4	452	40	34	17	7
Thoracoabdominal aorta*^1）^	50	0	0	13	174	28	114	26	10
Abdominal aortic aneurysm*^2）^	8,126	5,880	1,132	63	10,821	62	6,985	393	85
with renal artery reconstruction	357	269	47	11	20	12	337	36	7
with renal artery clamping	1,350	1,038	223	13	19	11	1,298	63	13

＊6) Debranch bypass surgery combined with two staged TEVAR is counted as one case of hybrid treatment. ＊7) Only for open surgery.

### 2) Abdominal aortic aneurysm (Tables 2-1 and 2-2)

**Figure 1:**
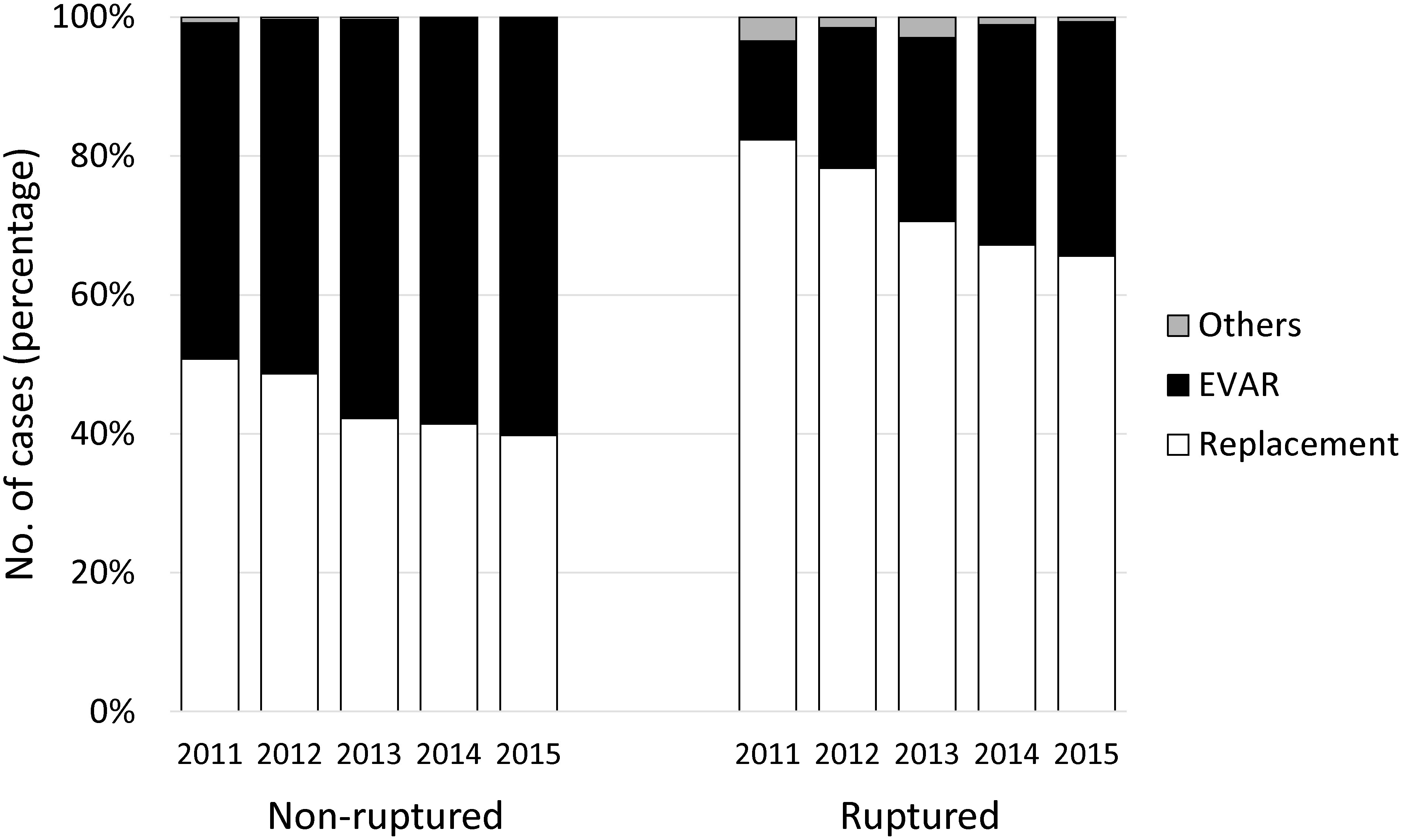
Fig. 1 Treatment procedure for non-ruptured and ruptured abdominal aortic aneurysm (AAA). Comparing year 2011, 2012, 2013, and 2014, proportion of EVAR selection was gradually increased in 2015.

In 2015, the total number of NCD-registered surgical cases of abdominal aortic aneurysm (including iliac artery aneurysm) was 18,907, which was increased from 15,745 in 2012, 16,694 in 2013, and 17,973 in 2014 (year-on-year increases of approximately 1,000). The surgical cases break down into 8,126 (43.0%) cases of replacement and 10,883 (57.6%) cases of stent-graft deployment (endovascular aneurysm repair; EVAR) (including hybrids). Since surpassing the majority in 2013, the number of EVAR cases has been continuously increasing (47.6% in 2012, 52.9% in 2013, and 55.7% in 2014). The number of replacement cases has almost flattened out, with the increases in EVAR directly equaling the increases in the total number (**Fig. 1**).

**Table:** Table 2-2 Abdominal aortic aneurysm mortality classified by treatment procedures

Procedure for aneurysm repair	Ruptured aneurysm			Non-ruptured aneurysm		
	Cases	30-day mortality	Hospital mortality	Cases	30-day mortality	Hospital mortality
Replacement	1,225	203	251	6,826	65	100
Exclusion with bypass	14	4	4	49	2	3
EVAR*^8）^	621	90	114	10,224	43	61
Hybrid	7	1	2	55	0	0

＊8) EVAR: endovascular aneurysm repair

Of all replacement cases, 1,350 required renal artery clamping (16.6%) and 357 required renal arterial reconstruction (4.4%). With the widespread adoption of EVAR, the cases of pararenal arteriopathy requiring renal artery clamping are expected to increase. In fact, the percentage has slightly yet steadily increased from 14.2% in 2012 to 15.4% in 2013 and 15.8% in 2014.

**Figure 2:**
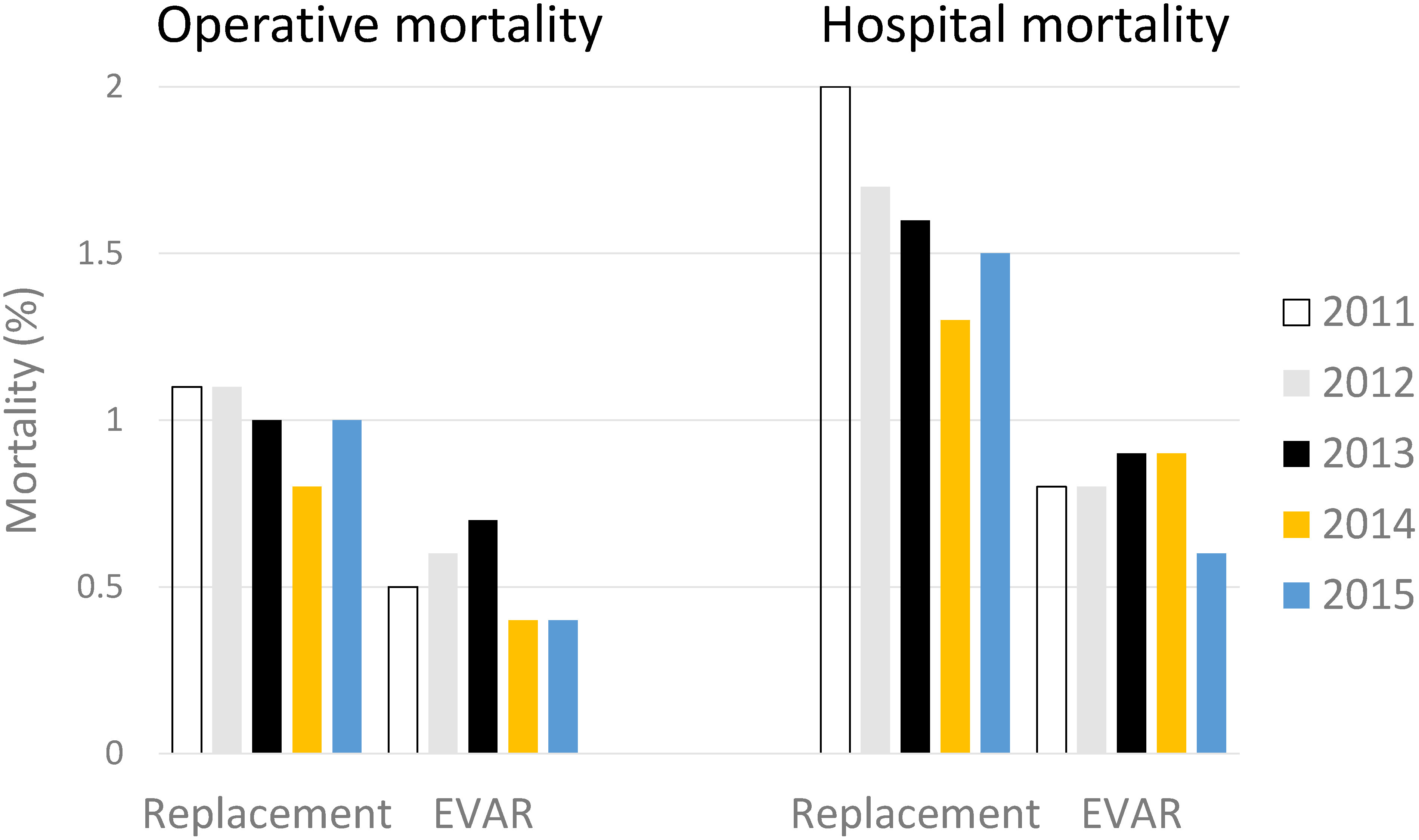
Fig. 2 Early clinical results of non-ruptured AAA in year 2015 comparing with those in year 2011, 2012, 2013, and 2014. Regarding the statistical difference of mortality rates between open repair (replacement) and EVAR, see main text.

Regarding the treatment results of non-rupture cases, the operative and the hospital mortalities from replacement were 1.0% and 1.5%, respectively, and those from EVAR (including special and hybrid techniques) were 0.4% and 0.6%, respectively (**Fig. 2**). When replacement involved renal artery clamping, the mortalities were aggravated to 1.9% and 3.1%, respectively. When reconstruction was added to these procedures, the mortalities were further aggravated to 2.2% and 4.4%, respectively.

EVAR: endovascular aneurysm repair

**Figure 3:**
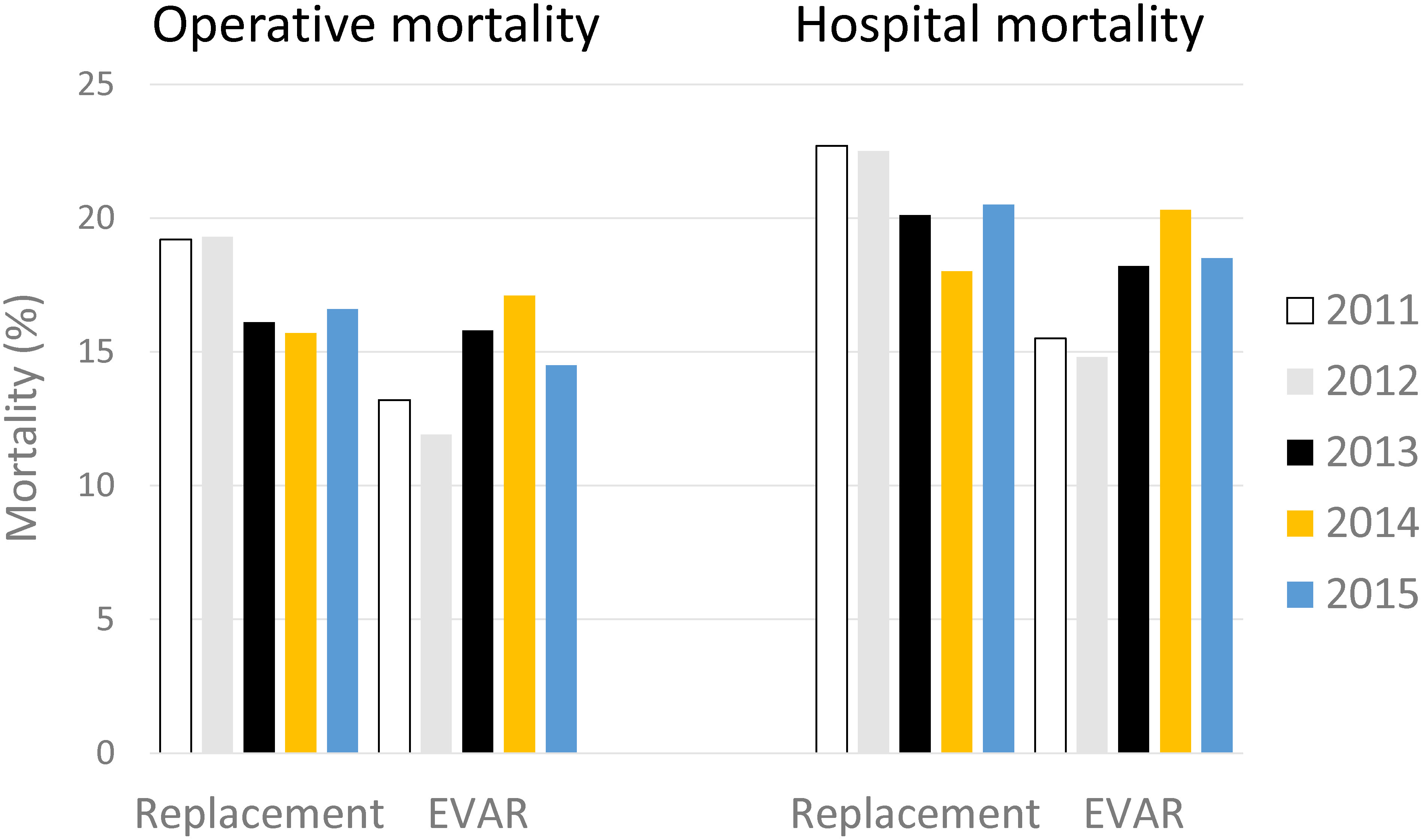
Fig. 3 Early clinical results of ruptured AAA in year 2015 comparing with those in year 2011, 2012, 2013, and 2014. Regarding the statistical difference of mortality rates between open repair (replacement) and EVAR, see main text.

The number of cases of rupture surgery was 1,850, with the operative and hospital mortalities being 16.0% and 19.9%, respectively. These results were almost identical to those obtained in 2014 (16.1% and 18.7%, respectively). EVAR was performed for 628 (33.6%) cases. Although the ratio of EVAR for rupture cases was increasing in the past few years, it slightly flattened out this year (14% in 2011, 20% in 2012, 25.5% in 2013, and 30.1% in 2014). The operative and hospital mortalities from EVAR for rupture cases were 14.5% and 18.5%, respectively. Although there was a slight year-on-year aggravation tendency (11.9% and 14.8% in 2012, 15.8% and 18.2% in 2013, and 17.1% and 20.3% in 2014), a slight improvement was observed from 2014 to 2015. Since the ratios of surgical modalities remained unchanged, surgical techniques might have improved (stabilized) at institutions that introduced EVAR for rupture cases (**Fig. 3**).

### 3) Peripheral artery aneurysm (Table 2-3)

Overall, 1,979 cases were registered. There were more male patients than female patients (with a ratio of 1,444 to 535). By region, most cases (857) concerned the lower limb arteries, followed by 731 cases concerning the abdominal visceral arteries, 373 cases concerning the upper limb arteries, and 50 cases concerning the aortic arch branching. The total number was 2,011; thus, it was inferred that 32 aneurysms simultaneously developed in multiple regions. By artery, 23.6% of the cases occurred in the femoral artery, 13.4% in the popliteal artery, and 9.8% in the brachial artery. In the “others” category of the abdominal visceral arteries (25.8%), the majority is considered to be an internal iliac arterial aneurysm. Therefore, modifications should be made to the registration method. Overall, 42.8% were symptomatic, and the most prevalent cause of illness was degenerative disease (65.3%). By surgical modality, 25.6% were ligation/dissection, 24.8% were replacement, 17.9% were coil embolization, 11.2% were stent grafting, and 7.8% were exclusion bypass. Given that the total number of surgical cases was 2,133, it was surmised that 7.2% received a combination of modalities or different modalities for simultaneously occurring aneurysms. Overall, the same trend as 2014 was observed in 2015.

**Table:** Table 2-3 Peripheral artery aneurysm

Aneurysm		Cases	Gender		Mortality		Ruptured aneurysm			Etiology					Treatment procedure						Graft material for open surgery			
			Male	Female	30-day mortality	Hospital mortality	Cases	30-d mortality	Hospital mortality	Degenerative	Vasculitis*^9)^	Infected	Traumas	Others	Replacement	Exclusion with bypass	Ligation/ resection	Stent graft	Coil embolization	Others	Polyester	ePTFE	Autogenous vessel	Others
Aortic arch branches																								
	Carotid	7	4	3	0	0	0	0	0	2	0	2	1	2	2	0	2	0	3	0	1	1	0	0
	Vertebral	0	0	0	0	0	0	0	0	0	0	0	0	0	0	0	0	0	0	0	0	0	0	0
	Subclavian	32	23	9	0	1	0	0	0	24	2	1	1	4	6	6	3	12	6	2	5	7	0	0
	Multiple in arch branches	3	2	1	0	0	0	0	0	2	0	0	0	1	0	0	1	1	1	0	0	0	0	0
	Others	8	7	1	2	2	1	1	1	3	0	2	1	2	3	0	3	1	2	0	0	3	0	0
Upper limb artery																								
	Axillar	16	8	8	0	1	0	0	0	12	1	1	0	2	11	1	4	0	0	1	2	5	5	0
	Brachial	197	106	91	5	8	3	0	0	42	0	26	56	73	34	10	104	0	1	56	3	16	25	2
	Forearm-hand	108	59	49	1	1	0	0	0	29	2	8	35	34	4	0	73	0	2	33	0	1	2	0
	Others	52	30	22	0	1	0	0	0	15	0	9	7	21	1	2	38	0	1	11	0	1	2	0
Visceral artery																								
	Celiac	38	30	8	2	1	0	0	0	26	1	4	1	6	11	2	8	6	13	3	5	1	5	1
	Hepatic	19	11	8	1	1	1	1	1	12	0	4	0	3	2	2	8	0	8	3	1	0	4	0
	Splenic	63	32	31	2	2	1	1	1	55	0	4	0	4	2	2	18	0	39	5	0	1	2	0
	Superior mesenteric	24	17	7	1	1	0	0	0	18	2	2	0	2	4	8	11	0	3	1	4	0	9	0
	Renal	68	42	26	0	1	0	0	0	60	0	2	0	6	14	3	15	3	29	11	1	0	12	0
	Others	519	444	75	5	6	3	0	0	465	5	6	5	38	86	10	50	194	253	15	76	14	5	0
Lower limb artery																								
	Femoral	475	373	102	10	15	1	0	0	235	3	41	75	121	191	24	155	11	7	100	93	92	33	0
	Popliteal	270	195	75	1	2	0	0	0	243	3	4	11	9	142	96	32	2	2	12	29	73	133	0
	Others	112	87	25	5	6	0	0	0	72	5	7	8	20	29	12	32	10	16	16	23	5	12	0
Total		1,979	1,444	535	35	48	10	3	3	1,292	24	118	200	345	530	169	546	238	382	268	236	216	240	3

＊9) Including TAO, Takayasu aortitis, collagen disease related vasculitis, Behcet disease, fibromuscular dysplasia. Abbreviations; Y-graft: Y-shape artificial graft, T-graft: straight artificial graft, Polyester: polyester artificial graft such as Dacron graft, ePTFE: expanded polytetrafluoroethylene graft

## 2. Revascularization for Chronic Arterial Occlusion (Table 3)

### 1) Arch branching, upper limb arteries, and abdominal visceral arteries (Table 3-1)

Compared with 2014, 2015 saw increases in cases concerning the carotid artery, subclavian artery, aortic arch branching multiple lesions, axillary artery to upper limb artery, and renal artery. Despite some fluctuations, no significant changes were noted in the other vertebral, celiac, and superior mesenteric arteries. In 2015, debranching associated with TEVAR/EVAR was added as a new item, and the following cases were registered: 39 cases of ascending aorta-brachiocephalic artery-left common carotid artery (-left subclavian artery) bypass, 125 cases of right axillary (subclavian)-left common carotid artery (-left subclavian artery) bypass+right common carotid-left common carotid artery bypass+left common carotid-left subclavian artery bypass, 172 cases of right axillary (subclavian)-left axillary (subclavian) bypass, and 21 cases of abdominal aorta-superior mesenteric-renal artery bypass. However, the increase in the cases of carotid artery-related surgery was mainly attributed to the increase in the cases of carotid artery-subclavian artery bypass and axillo-axillary artery bypass (possibly debranching). Since a debranching-related item was newly introduced in 2015, such cases were registered separately from cases applicable to the other existing items. Therefore, caution is required for increases or decreases in the number of cases in each item. However, the number of cases of arch branching bypass (possibly related to debranching) has been greatly increasing per year. This may signify that stent-graft deployment for anatomically complex aortic aneurysms has been performed increasingly (**Table 3-6**).

**Table:** Table 3 Reconstruction for chronic arterial occlusive diseases*^10)^ Table 3-1 Arterial reconstruction for aortic arches

Aortic branches	Cases	Gender		Mortality	Background	Etiology					Revascularization procedures												Graft materials*^14)^				Previous reconstruction					Revision reason							
		Male	Female	30-day mortality	Dialysis	ASO	TAO	Vasculitis*^11)^	Takayasu arteritis	Others	CAS		CEA		PTA/ stent*^13)^	Replacement	Visceral artery bypass	Internal iliac artery bypass	Anatomical bypass	Carotid-subclavian bypass	Axillo-axillar bypass	Others	Polyester	ePTFE	Autogenous veins	Others	None	Once	Twice	Three times and more	Unclear	Host artery stenosis/ occlusion	Graft stenosis	Graft occlusion	EVT stenosis	EVT occlusion	Stent graft-caused stenosis/occlusion	Poor symptom recovery	Other
											Cases	Brain complication*^12)^	Cases	Brain complication*^12)^	Cases																								
Carotid artery	174	146	28	4	7	63	0	0	1	12	5	0	54	4	13	1	0	0	13	88	73	7	25	93	4	2	157	13	1	2	1	4	2	0	2	0	1	0	8
Vertebral artery	6	5	1	0	0	1	0	0	0	0			0	0	1	0	0	0	0	2	3	2	2	3	1	1	6	0	0	0	0	0	0	0	0	0	0	0	0
Subclavian artery	353	266	87	7	28	115	0	2	5	21			0	0	77	1	1	0	14	113	203	27	67	210	8	3	320	22	3	6	2	6	0	11	6	4	0	1	6
Multiple lesions of arch branches	13	8	5	0	0	7	0	0	0	1			0	0	0	0	0	0	0	3	12	1	9	10	0	1	12	1	0	0	0	0	0	0	0	0	0	1	0
Upper limb including axillar artery	111	80	31	1	61	77	2	1	1	18			0	0	38	3	3	0	18	4	10	40	12	20	20	5	76	21	4	4	6	9	7	5	3	0	0	2	4
Celiac/Superior mesenteric artery	79	57	22	5	9	51	1	1	0	19			0	0	22	5	31	5	6	1	1	12	20	16	11	4	67	10	1	1	0	4	0	1	2	0	0	0	5
Renal artery	129	97	32	2	6	109	0	0	0	14			0	0	110	3	9	0	2	0	0	6	10	5	3	1	110	15	1	2	1	5	2	0	7	1	1	0	5
Others	301	240	61	9	29	38	0	0	0	11			0	0	40	2	17	6	39	84	116	64	112	145	6	6	288	9	0	2	2	5	1	0	0	0	0	1	7
Total	915	695	220	19	120	449	3	4	7	95	5	0	54	4	290	15	53	11	70	147	238	142	186	321	49	21	789	88	10	17	11	33	12	17	20	5	2	5	32

＊10) Bypass surgery combined with endovascular treatment is counted in both bypass category (Table 3-2) and endovascular category (Table 3-5). ＊11) Including TAO, Takayasu arteritis, Coarctation of aorta, collagen disease related vasculitis, Behcet disease, fibromuscular dysplasia. ＊12) Postoperative irreversible brain complication. ＊13) Including percutaneous transluminal angioplasty (PTA), stent, and other endovascular means such as catheter atherectomy. ＊14) Only for open surgery.

**Table:** Table 3-2 Arterial reconstruction for chronic lower limb ischemia

From aorta to lower limb arterial systems	Cases	Gender		Mortality	Dialysis cases	Etiology					Graft materials				Previous reconstruction					Revision reason							
		Male	Female	30-day mortality		ASO	TAO	Vasculitis	Takayasu arteritis	Others	Polyester	ePTFE	Autogenous veins	Others	None	Once	Twice	Three times and more	Unclear	Host artery stenosis/ occlusion	Graft stenosis	Graft occlusion	EVT stenosis	EVT occlusion	Stent graft-caused stenosis/occlusion	Poor symptom recovery	Other
Aorto-aortic bypass	41	29	12	0	4	35	0	0	3	3	28	10	4	0	33	7	1	0	0	3	0	1	1	1	0	1	1
Infrarenal aortic reconstruction (suprarenal clamp)	37	33	4	0	1	34	0	1	0	2	36	2	2	1	33	3	0	0	1	0	0	1	1	1	0	0	0
Aorto-femoral bypass*^15)^	562	445	117	4	33	545	0	2	1	13	417	161	26	7	470	61	17	12	2	18	10	25	8	11	6	6	10
Femoro-popliteal (above the knee) bypass	1,810	1,327	483	13	254	1,791	3	3	0	13	293	1,260	337	27	1,332	313	86	73	6	120	30	105	30	90	12	44	53
Infrapopliteal arterial bypass	1,872	1,380	492	33	605	1,810	21	13	0	28	92	453	1,386	77	1,117	419	138	184	14	185	43	178	61	115	11	107	59
Femoro-popliteal (below the knee) bypass	726	516	210	12	168	709	2	3	0	12	51	332	393	25	450	162	42	65	7	60	11	86	17	45	9	28	24
Femoro-crural/pedal bypass*^16)^	1,194	900	294	22	454	1,148	19	11	0	16	42	144	1,038	52	701	263	98	125	7	130	32	94	44	75	3	80	35
Others	98	74	24	3	17	91	2	0	0	5	38	43	28	0	65	18	5	9	1	8	7	8	1	3	2	5	1
Total	4,257	3,159	1,098	50	882	4,147	25	17	4	63	826	1,831	1,720	108	2,928	798	243	266	22	328	87	305	98	218	29	160	117

＊15) Including aorto-iliac bypass or ilio-femoral bypass. ＊16) Including popliteal-crural (or pedal) bypass.

**Table:** Table 3-3 Extra-anatomical bypass*^17)^

Extra-anatomical bypass	Cases	Gender		Mortality	Dialysis cases	Etiology			Graft materials				Previous reconstruction					Revision reason							
		Male	Female	30-day mortality		ASO	TAO	Others	Polyester	ePTFE	Autogenous veins	Others	None	Once	Twice	Three times and more	Unclear	Host artery stenosis/occlusion	Graft stenosis	Graft occlusion	EVT stenosis	EVT occlusion	Stent graft-caused stenosis/ occlusion	Poor symptom recovery	Other
Carotid-subclavian bypass	147	115	32	7	10	16	0	6	41	103	3	2	137	8	1	0	1	0	1	4	0	1	1	0	3
Axillo-axillar bypass	238	186	52	6	15	51	0	15	45	198	1	1	228	7	1	1	1	2	0	5	0	1	1	1	1
Axillo-femoral bypass*^18)^	372	249	123	8	46	346	2	24	129	230	21	16	289	62	9	8	4	13	8	26	8	8	4	2	12
Femoro-femoral crossover bypass	825	661	164	9	85	783	4	27	211	591	61	16	617	151	26	27	4	41	17	57	8	35	12	19	19
Others	114	92	22	2	10	104	0	3	26	85	11	1	72	26	8	8	0	10	2	16	1	3	2	1	8
Total	1,592	1,224	368	27	157	1,269	5	73	427	1,124	94	35	1,252	243	45	43	9	64	25	105	16	47	18	23	42

＊17) Cases underwent extraanatomical bypass because of graft infection should not be included this category. Those cases are listed in vascular complication (Table 6). ＊18) A case underwent axillo-femoro-femoral crossover bypass is counted as one case. A case combined with additional contralateral side of axillo-femoral bypass as second staged surgery is counted as 2 cases.

**Table:** Table 3-4 Thromboendarterectomy*^19)^ for chronic lower limb ischemia

Thromboendarterectomy	Cases	Gender		Mortality	Dialysis cases	Etiology			Previous reconstruction					Revision reason							
		Male	Female	30-day mortality		ASO	TAO	Others	None	Once	Twice	Three times and more	Unclear	Host artery stenosis/occlusion	Graft stenosis	Graft occlusion	EVT stenosis	EVT occlusion	Stent graft-caused stenosis/occlusion	Poor symptom recovery	Other
Aorto-iliac lesion	55	44	11	0	8	52	0	2	36	10	3	5	1	4	3	2	0	4	1	0	4
Femoro-popliteal lesion	960	705	255	7	233	952	0	8	730	148	44	33	5	102	7	22	29	27	5	20	21
Others	476	356	119	16	119	423	1	49	267	103	42	53	11	25	37	27	10	19	4	32	48
Total	1,466	1,086	379	23	353	1,404	1	57	1,012	258	88	91	17	130	47	50	39	49	10	52	72

＊19) Including patch plasty.

**Table:** Table 3-5 Endovascular treatment for chronic lower limb ischemia*^13)^

Endovascular treatment	Cases	Gender		Mortality		Dialysis cases	Etiology			Previous reconstruction					Revision reason							
		Male	Female	30-day mortality	Hospital mortality		ASO	TAO	Others	None	Once	Twice	Three times and more	Unclear	Host artery stenosis/occlusion	Graft stenosis	Graft occlusion	EVT stenosis	EVT occlusion	Stent graft-caused stenosis/ occlusion	Poor symptom recovery	Other
Aorto-iliac lesion*^20)^	3,284	2,646	638	23	31	394	3,218	3	54	2,656	404	114	93	17	221	33	39	151	43	48	38	61
Femoro-popliteal lesion*^20)^	3,229	2,194	1,035	36	77	904	3,199	4	25	1,944	683	256	317	29	410	129	64	354	143	40	85	56
Infrapopliteal-ankle lesion*^20)^	1,803	1,181	622	34	84	878	1,776	3	23	974	360	156	286	27	264	97	47	206	110	4	61	32
Others	197	128	69	2	6	83	192	0	5	32	64	22	77	2	19	98	19	15	3	5	1	4
Total (number of regions underwent EVT)*^20)^	7,441	5,458	1,983	73	155	1,857	7,321	9	100	4,943	1,349	464	618	67	767	312	143	620	251	87	171	139
Total (number of limbs underwent EVT)*^21)^	6,417	4,795	1,622	54	117	1,473	6,304	8	94	4,304	1,195	380	479	59	631	269	119	519	205	78	158	125

＊20) When endovascular treatment performed for multiple regions, the case should be counted in each regions (If a case underwent endovascular treatment in both aorto-iliac and femoro-popliteal region, this case can be counted one in aorto-iliac, and one in femoro-popliteal region). ＊21) Counting the patients number not treated regions. When a case underwent endovascular treatment in multiple region, the case is counted as one case. Abbreviations; ASO: arteriosclerosis obliterans; TAO: thromboangiitis obliterans (Buerger’s disease); CAS: carotid artery stenting; CEA: carotid endarterectomy; PTA: percutaneous transluminal angioplasty; EVT: endovascular treatment; IIA: internal iliac artery

**Table:** Table 3-6 Debranch for TEVAR or EVAR

Debranch for TEVAR or EVAR	Cases
Ascending aorta-brachiocephalic-left common carotid (-left subclavian) arterial bypass	39
Right axillar-left common carotid (-left axillary) arterial bypass	125
Right common carotid-left common carotid (-left subclavian) arterial bypass	
Left common carotid-left subclavian arterial bypass or transposition	
Right axillar (subclavian)-left axillar (subclavian) arteril bypass	172
Abdominal aorta (iliac) (-celiac)-superior mesenteric-renal arterial bypass	21

### 2) Anatomical bypass for the aorta-lower limb artery region (Table 3-2), extra-anatomical bypass (Table 3-3), and endovascular treatment (Table 3-5)

#### Aortoiliac area

**Figure 4:**
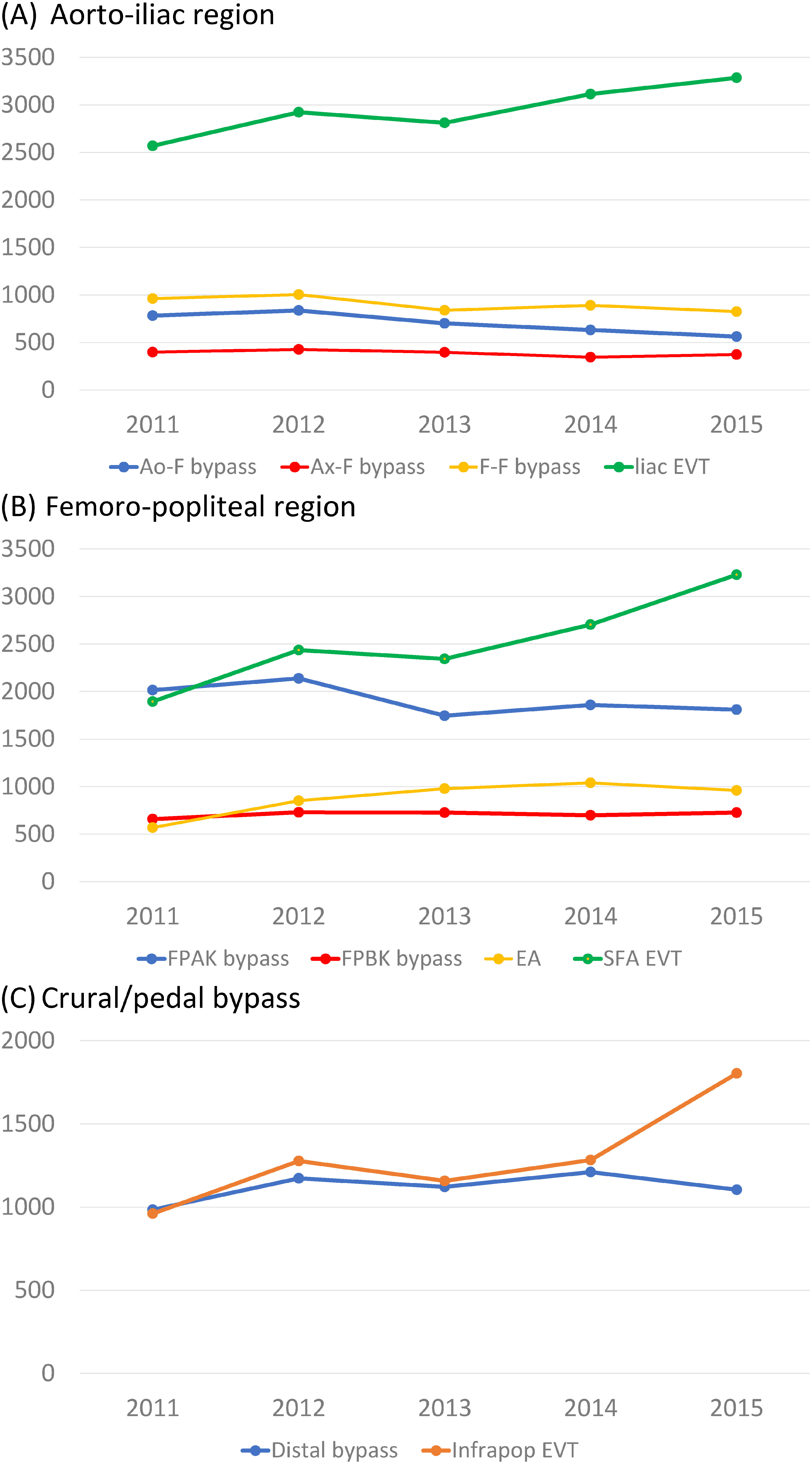
Fig. 4 The annual trends of the number of arterial reconstructions in aorto-iliac (**A**), femoro-popliteal (**B**), and crural/pedal region (**C**), comparing open repair and endovascular treatment.

Ao-F: aorto-femoral; Ax-F: axillo-femoral; F-F: femoro-femoral crossover; EVT: endovascular treatment; FPAK: femoro-popliteal (above the knee); FPBK: femoro-popliteal (below the knee); SFA: superficial femoral artery; EA: endarterectomy

The number of cases performing anatomical bypass for aortoiliac area lesions decreased from 733 in 2014 to 640 in 2015 (approximately 15% decrease). Nevertheless, there were no changes in the surgery items, including synthetic vascular grafts. Regarding extra-anatomical revascularization procedures, as represented by axillo-femoral artery bypass and femoro-femoral artery bypass, the numbers of cases increased from 345 and 890 in 2014 to 372 and 825 in 2015, respectively. Hence, the number of cases slightly increased in the former and decreased in the latter. However, the total number did not change significantly, and the details remained unchanged. The rate of revascularization in the past was 13% for anatomical bypass, which was less frequent than 23% for extra-anatomical bypass. This ratio was unchanged from the previous years. The decrease in the cases of anatomical reconstruction was nearly equal to the increase in the cases of endovascular treatment; thus, it was inferred that the actual number of revascularization procedures was not greatly changed in this region (**Fig. 4A**).

### 3) Superficial femoral artery

The number of cases performing femoral above-knee popliteal artery bypass was almost unchanged from 1,859 in 2014 to 1,810 in 2015. Overall, 26% of the patients had a previous history of revascularization, and 70% of the synthetic vascular grafts were made of ePTFE, whereas 19% used autogenous veins (**Fig. 4B**).

### 4) Revascularization below the knee joint

Of the revascularization cases performed below the knee joint, the numbers of cases of femoral below-knee popliteal artery bypass and femoro-crural/pedal artery bypass were 699 and 1,210 cases in 2014 and 726 and 1,194 cases in 2015, respectively. The number of cases of revascularization below the knee joint (including cases of distal bypass) did not change significantly from 2014 (**Figs. 4B** and **4C**). Of the cases of femoro-crural/pedal artery bypass, 39% required dialysis, which was a slight increase from the previous year. This suggests a slight increase in bypass cases for more serious patients. Moreover, 40% had a history of revascularization, which was more than the history of above-knee bypass. For vascular graft, 86% used autogenous veins.

### 5) Thromboendarterectomy (Table 3-4)

The number of cases performing thromboendarterectomy in the lower limb artery system in the femoropopliteal artery region was 1,039 in 2014, which decreased by 8% to 960 in 2015. However, since this report included replaced items, the number of cases using other modalities greatly increased from 121 in 2014 to 476 in 2015. These cases probably included several instances of femoral reconstruction with graft. Therefore, it was likely that more lesions in the common femoral artery, which are difficult to address with endovascular treatment, were treated as a whole (**Fig. 4B**).

### 6) Endovascular treatment (Table 3-5)

The total number of cases performing endovascular treatment increased by approximately 1,000 (17%) from 2014, 25% of which were performed for dialysis cases. While an almost equal number of revascularization procedures (bypass and thromboendarterectomy) were performed compared with the numbers in 2014, there was a marked increase in cases performing endovascular treatment. In particular, endovascular treatment was increasingly being applied to occlusive arterial diseases. In fact, endovascular treatment accounts for 46% of all the revascularization procedures for chronic arterial occlusion. In the crural artery region, particularly, 1,803 cases underwent endovascular treatment in 2015, which was a marked 40% increase from 1,283 cases in 2014. The increase rate was 19% in the femoropopliteal artery region, whereas the number was almost unchanged from 2014 in the iliac artery region. In the regions below the inguinal ligament, the number of endovascular treatment cases increased significantly (**Figs. 4A–4C**).

## 3. Revascularization for Acute Arterial Occlusion (Table 4)

The number of acute arterial occlusion cases excluding vascular trauma was 4,779. The lesions below the abdominal aorta accounted for approximately 80% overall, with the cases of thrombosis and embolism being half each. This result was consistent with that of the previous years. Given that the total number of obstruction cases in all regions was 5,527, 748 (13.5%) probably had obstruction in multiple sites, and this ratio was also consistent with that of the previous years. The number of cases performing thrombolytic therapy (which was added as a new item in 2013) was 62 (70 in the previous year). Overall, the percentage of percutaneous transluminal angioplasty (PTA)±stent cases was 14.9%, showing a year-on-year increasing trend (12.6% in the previous year). The implementation rate of intravascular treatment (PTA±stent; thrombolysis) was 25.9% in the abdominal aorta-iliac artery region (23.3% in the previous year) and 15.8% in the femoropopliteal artery region (13.1% in the previous year).

**Table:** Table 4 Revascularization for acute arterial occlusive disease*^22)^

Obstructive artery*^23)^	Cases	Gender		Mortality		Etiology			Procedure						Graft materials for open surgery			
		Male	Female	30-day mortality	Hospital mortality	Embolism	Thrombosis*^24)^	Others	Thrombectomy±patch*^25)^	Bypass	Replacement	PTA±stent	Thrombolysis	Other	Autogenous vessel	Polyester	ePTFE	Others
Carotid artery	10	4	6	1	1	4	2	4	2	5	0	1	0	2	0	3	2	0
Subclavian artery	53	31	22	1	2	18	26	9	38	12	1	6	0	0	0	5	9	0
Axillar artery	85	39	46	5	6	34	45	6	61	14	2	8	0	4	4	5	8	0
Brachial artery	699	320	379	24	29	297	379	23	606	18	2	35	4	68	13	13	9	3
Celiac/superior mesenteric artery	112	78	34	16	20	43	30	39	46	33	2	19	2	14	29	8	4	2
Renal artery	37	23	14	4	5	11	6	20	8	7	0	25	1	1	1	3	5	0
Abdominal aorta-iliac artery	829	558	271	102	124	281	436	112	487	252	21	205	10	40	10	122	157	12
Femoro-popliteal artery	2,601	1,599	1,002	221	283	1,102	1,400	99	2,070	324	19	377	35	142	139	129	216	19
Crural artery	795	488	305	72	106	346	433	16	637	61	4	145	23	64	44	25	34	4
Pedal artery*^26)^	55	31	24	11	15	26	29	0	43	8	0	9	3	5	7	2	2	1
Others	251	184	67	9	14	48	177	26	183	18	1	35	4	39	14	9	12	0
Total	4,779	2,940	1,837	367	476	1,855	2,592	332	3,546	652	48	714	62	345	233	292	393	36

＊22) Cases with non-traumatic acute arterial occlusion are listed in this table. Please see Table 5-1 for acute arterial occlusion by trauma. ＊23) The most proximal occluded artery name is described in case whose primary occluded artery could not be identified. ＊24) Cases with acute worsening occlusion of chronic arterial occlusive disease are excluded. Treatment for those cases are listed in Table 3. ＊25) If either thrombectomy or patch plasty is performed, cases are listed in this section. ＊26) Including acute occlusion of dorsalis pedis or planter artery.

The use rate of synthetic vascular grafts in bypass surgery was 68.6% (67.6% in the previous year) in the femoropopliteal artery and 55.1% (54.8% in the previous year) in the crural artery regions. Even in the crural artery, synthetic vascular grafts were used in more than half the cases of acute arterial occlusion, and this was consistent with the results of previous years.

The operative and hospital mortalities were 12.3% and 15.0% in the abdominal aorta-iliac artery region, 8.5% and 10.9% in the femoropopliteal artery region, 9.1% and 13.3% in the crural artery region, and 20.0% and 27.3% in the pedal artery region, respectively. Compared with the cases performing elective revascularization, prognosis was clearly poor. Particularly, the operative and hospital mortalities for pedal arterial occlusion significantly increased from those of the previous year (5.1%/15.3%).

Of the 112 cases in the abdominal artery-superior mesenteric artery region, the operative mortality was 14.3% and the hospital mortality was 17.9%, showing an extremely poor prognosis similar to those of the previous years. The implementation rate of endovascular treatment was only 18.8% in this region, with surgical therapies such as thrombectomy and bypass surgery being the primary modalities.

## 4. Treatment for Vascular Trauma (Table 5)

**Figure 5:**
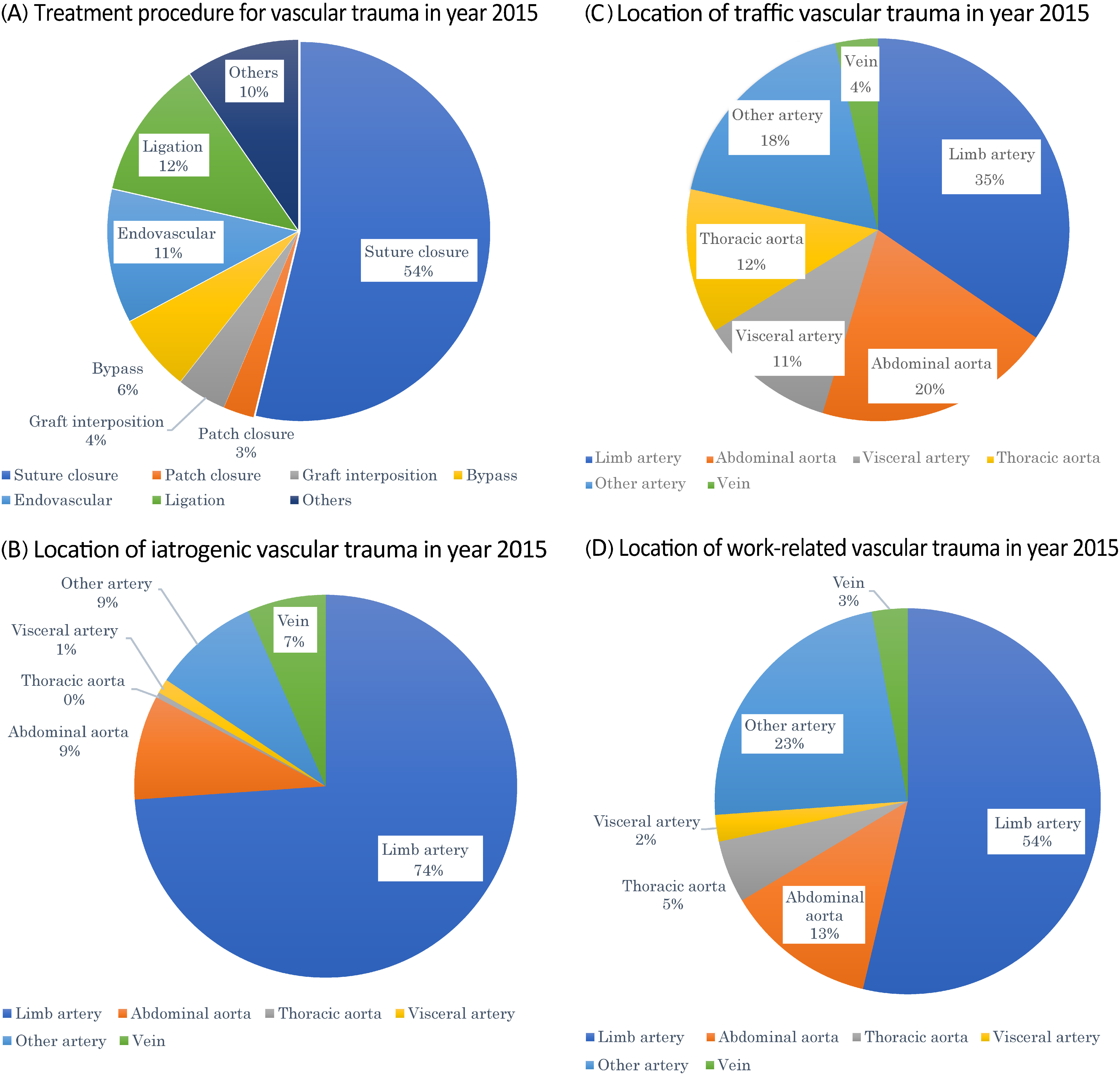
Fig. 5 Treatment procedure and location of vascular trauma in year 2015. Operation mode (**A**), location vascular trauma by iatrogenic (**B**), by traffic accident (**C**), and work-related accident (**D**).

**Table 5** lists the sites of vascular trauma, causes of injury, surgical modalities, and types of vascular grafts used as registered in the NCD in 2015. The total number of cases of arterial/venous trauma was 2,313. The most prevalent cause of vascular trauma was iatrogenic, accounting for 1,561 (67%) cases, followed by traffic accidents in 134 (6%) cases and occupational hazards in 129 (6%) cases. The most prevalent site of vascular injury was the lower limb arteries, accounting for 1,095 (47%) cases, followed by 425 (18%) cases in the upper limb arteries and 246 (10%) cases in the abdominal-iliac artery. Therapeutic modalities were registered in 2,426 cases. By modality, direct suture was used in 1,305 (54%) cases, ligation in 286 (12%) cases, and endovascular treatment in 276 (11%) cases (**Fig. 5A**). Vascular grafts were used in 326 cases, and 47% of the vascular grafts used were autogenous vessels.

**Table:** Table 5 Treatment for vascular trauma Table 5-1 Arterial trauma

Injured artery	Cases	Gender		Mortality		Cause of trauma				Procedure							Status of injured artery*^27)^						Prosthesis			
		Male	Female	30-day mortality	Hospital mortality	Traffic accident	Labor accident	Iatrogenic	Others	Direct closure	Patch plasty	Replacement	Bypass	Endovascular	Ligation	Others	Obstruction/stenosis*^28)^	Bleeding without specification*^29)^	GI fistula	Non-GI fistula	Pseudo-aneurysm	Others	Autogenous vessel	Polyester	ePTFE	Others
Carotid artery	29	19	10	6	6	2	0	16	11	12	1	0	2	5	5	6	1	16	2	2	3	5	1	0	2	0
Subclavian artery	56	36	20	5	9	4	2	41	9	28	2	1	2	12	8	5	7	30	0	0	7	12	0	0	4	1
Axillar artery	29	17	12	0	3	1	2	17	9	13	0	0	9	3	1	6	9	10	1	0	5	5	3	3	3	0
Brachial artery	340	200	140	6	12	7	11	274	48	232	2	8	22	8	44	35	42	66	0	6	197	42	26	0	5	1
Descending aorta (thoracic/ thoracoabdominal)	45	32	13	12	12	17	7	7	14	6	0	2	1	22	6	9	5	22	5	1	8	6	0	2	1	0
Celiac/ superior mesenteric artery	36	25	11	2	3	13	2	13	8	10	0	2	5	18	2	0	12	18	2	0	3	3	3	1	2	0
Renal artery	19	16	3	0	0	3	1	6	9	2	0	1	2	8	0	6	6	11	0	0	3	2	1	0	2	0
Abdominal aorta-iliac artery	246	147	99	20	26	28	17	137	64	54	8	30	34	107	12	25	47	105	9	11	22	60	9	38	26	1
Femoro-popliteal artery	1,045	655	390	141	196	30	44	798	173	754	38	40	70	46	64	69	120	270	2	16	360	318	81	22	43	4
Crural artery	50	39	11	1	2	6	13	19	12	18	2	2	13	6	5	6	16	15	0	0	15	6	13	0	3	0
Others	302	179	123	21	28	23	31	140	108	109	5	6	9	42	91	55	27	143	3	18	50	63	9	3	3	3
Total	2,167	1,345	822	213	294	129	125	1,459	454	1,233	58	90	154	269	238	216	281	696	23	54	669	515	142	65	86	10

**Table:** Table 5-2 Venous trauma*^27)^

Injured veins	Cases	Cause of trauma				Procedure							Prosthesis			
		Traffic accident	Labor accident	Iatrogenic	Other	Direct closure	Patch plasty	Replacement	Bypass	Endovascular	Ligation	Others	Autogenous vessel	Polyester	ePTFE	Others
Superior vena cava	6	0	0	4	2	2	2	1	0	0	1	2	0	1	0	2
Inferior vena cava	10	0	1	5	4	6	1	1	0	0	1	1	1	0	1	0
Brachiocephalic-subclavian vein	11	1	1	9	0	6	0	1	0	2	3	1	0	1	0	0
Iliac-femoral-popliteal vein	62	1	1	52	8	34	2	7	2	4	10	8	7	2	2	0
Others	60	3	1	33	23	24	0	3	4	1	33	8	3	0	4	0
Total	146	5	4	102	35	72	5	12	6	7	48	18	11	3	7	2

＊27) Iatrogenic pseudoaneurysm in endovascular treatment is listed in Table 5-1. ＊28) Including arterial dissection. ＊29) Without GI fistula or non-GI fistula. Cases with vessel injury involving both vein and accompanying artery are listed in Table 5-1. Abbreviation; GI: gastro-intestinal

### 1) Iatrogenic vascular trauma (Fig. 5B)

Of the 1,561 cases and 1,571 sites of iatrogenic vascular trauma, the most prevalent site was the lower limb arteries (817 cases; 52%), followed by the upper limb arteries (332 cases; 21%). Therefore, the lower and upper limb arteries combined accounted for 74%, most of which probably occurred as complications of paracentesis associated with endovascular catheterization and treatment.

### 2) Traffic accident injury (Fig. 5C)

Of the 134 cases and 139 sites of traffic accident injuries, the most prevalent site was the upper and lower limb arteries (48 cases; 35%), followed by the abdominal aorta-iliac artery (28 cases; 20%), the descending aorta-thoracoabdominal aorta (17 cases; 12%), and visceral arteries (16 cases; 12%). Situated close to the body surface, blood vessels in the four extremities are frequently subjected to external force and, hence, are susceptible to injury. However, unlike the other causes, the prevalent sites of traffic accident injuries include the thoracic/abdominal aortic regions that are protected by the rib cage and abdominal wall. This is probably because traffic accidents involve high-energy trauma due to sudden collision and deceleration.

### 3) Occupational hazard injury (Fig. 5D)

Occupational hazard injuries mainly include accidents at work, such as falling from high place and getting stuck in working machinery. Overall, 129 cases and 134 sites were registered. By site, 72 (54%) cases occurred in the arteries in the four extremities, which were close to the body surface and subject to external force.

### 4) Summary

This report presented an overview of the registration status of vascular trauma cases in the NCD database in 2015. Compared with 2014, the overall number of registered cases slightly increased. Nevertheless, there were no significant differences in the causes/sites of trauma, types of vascular grafts, and therapeutic modalities.

## 5. Surgery for Revascularization Complications (Table 6)

As with reports of the previous years (up to 2014), the number of registered cases concerning the chest to thoracoabdominal region was small. The number of revascularization complications in this region could not be examined.

**Table:** Table 6 Revascularization for vascular complication after revascularization Table 6-1 Graft infection

Position of infected graft	Cases	Mortality		Status of infected graft				Procedure for graft infection			Material for revision or redo surgery				
		30-day mortality	Hospital mortality	Sepsis	Graft-GI fistula*^31)^	Graft-skin fistula*^31)^	Others	In-situ replacement	Extra-anatomical bypass	Others	Polyester	ePTFE	Autogenous vessel	Cryo-preserved homograft	Others
Descending thoracic aorta	3	0	0	2	1	0	0	0	0	2	1	1	0	0	0
Thoracoabdominal aorta	18	1	2	6	7	4	4	8	0	6	4	5	0	0	3
Abdominal aorta-iliac artery	57	5	10	23	22	6	15	21	0	15	25	14	6	0	3
Abdominal aorta-femoral artery	51	5	9	16	7	17	13	11	0	29	10	10	5	0	3
Femoro-distal artery	118	7	11	32	3	56	33	22	0	75	10	33	24	0	2
Others*^30)^	278	16	26	53	6	114	118	26	0	212	15	79	28	0	13
Total	525	34	58	132	46	197	183	88	0	339	65	142	63	0	24

＊30) Cases with graft infection involving aortic arch branch or upper limb artery are listed on this column. ＊31) Including anastomotic disruption. Abbreviation; GI: gastrointestinal

### 1) Vascular graft infection (Table 6-1)

As vascular graft infection, 525 cases were registered. 53.0% of which were the others region, including the arch branching and upper limb artery. In this region, the most prevalent condition of infection is the cutaneous fistula of vascular grafts, many of which were inferred to be infection in the shunts for dialysis. 22.5% of graft infection were femoro-distal artery. The overall operative mortality was 6.5%, and in-hospital mortality was 11.0%.

### 2) Arterial aneurysm in anastomotic sites (non-infectious) (Table 6-2)

There were 164 cases reported as aneurysm in anastomotic sites. By region, the most prevalent was the femoral artery, followed by the abdominal aorta, axillary artery-upper limb artery, and iliac artery. In the peripheral region beyond the abdominal aorta, arterial sclerosis was the most prevalent cause of illness.

**Table:** Table 6-2 Anastomotic aneurysm*^32)^

Location of anastomotic aneurysm	Cases	Mortality	Cause of aneurysm treated at the primary operation					Repair procedure				Material for repair surgery			
		30-day mortality	Degenerative	Takayasu arteritis*^33)^	Other vasculitis*^34)^	Infection	Others	Replacement	Exclusion and bypass	Stent graft	Others	Polyester	ePTFE	Autogenous vessel	Others
Aortic arch branch	2	0	2	0	0	0	0	0	0	0	2	1	0	0	0
Upper limb artery including axillar artery	28	0	3	0	0	2	23	8	2	1	18	1	5	8	2
Thoracic aorta	5	0	2	0	0	0	3	0	1	2	2	1	0	0	0
Splanchnic artery	5	1	3	0	0	1	1	2	0	2	1	3	0	0	1
Renal artery	3	0	1	0	0	0	2	0	0	0	3	0	0	1	0
Abdominal aorta	36	1	28	1	2	1	4	11	0	20	5	16	7	0	3
Iliac artery	23	4	15	0	1	1	6	4	1	10	8	9	3	0	1
Femoral artery	50	3	30	0	0	7	13	20	3	2	25	11	13	4	1
Popliteal or more distal lower limb artery	18	0	8	0	1	1	8	3	2	0	13	1	1	6	0
Total	164	9	88	1	3	12	60	47	9	32	77	41	29	19	7

＊32) Cases with infected pseudoaneurysm located at the anastomotic site to the artificial graft are listed in Table 6-1. ＊33) Including the atherosclerotic aneurysm. ＊34) Including TAO, collagen disease, Behcet disease, and fibromuscular dysplasia.

### 3) Autogenous vascular graft aneurysm (Table 6-3)

According to the report of autogenous vascular graft aneurysm, there were 21 cases in the upper and 39 cases in the lower limb arteries. The abdominal visceral artery was not reported as a site. By modality, 28.6% of the cases used replacement/bypass surgery in the upper and 25.6% in the lower limb arteries. The other regions were the most prevalent, but the details remained unclear.

**Table:** Table 6-3 Autogenous graft aneurysm

Revascularization area	Cases	Mortality	Repair procedure		
		30-day mortality	Replacement	Bypass	Others
Visceral artery	0	0	0	0	0
Upper limb artery	21	0	3	3	15
Lower limb artery	39	0	4	6	29
Others	11	1	3	2	6
Total	71	1	10	11	50

### 4) Degradation of vascular grafts (Table 6-4)

In 2015, 97 cases of vascular graft degradation were registered, which was a considerable increase from 52 in 2014. By initial modality, the number of replacement cases increased from 19 to 29, bypass surgery cases from 19 to 46, and stent-graft surgery from 3 to 6 (2014 vs. 2015). Degradation of polyester and ePTFE was also reported; however, the degradation rate could not be calculated because the statistical parameter was unknown.

**Table:** Table 6-4 Graft degeneration

Revascularization	Cases	Mortality	Initial revascularization procedure				Degenerative material			Repair procedure					Graft material		
		30-day mortality	Replacement	Bypass	Stent graft	Others	Polyester	ePTFE	others	Replacement	Bypass	Stent graft	Patch plasty	Others	Polyester	ePTFE	Others
Descending thoracic aorta	0	0	0	0	0	0	0	0	0	0	0	0	0	0	0	0	0
Thoracoabdominal aorta	2	0	1	1	1	0	1	1	0	1	0	1	0	0	1	1	0
Abdominal aorta-femoral artery	31	0	12	11	5	3	24	5	2	9	11	3	2	7	11	13	1
Femoro-popliteal artery	21	0	2	17	0	2	7	10	4	4	10	0	1	8	5	5	7
Others	43	1	14	17	0	12	10	14	19	9	9	3	3	19	9	11	10
Total	97	1	29	46	6	17	42	30	25	23	30	7	6	34	26	30	18

## 6. Venous Operation (Table 7)

### 1) Varicose veins in the lower extremities (Table 7-1)

**Figure 6:**
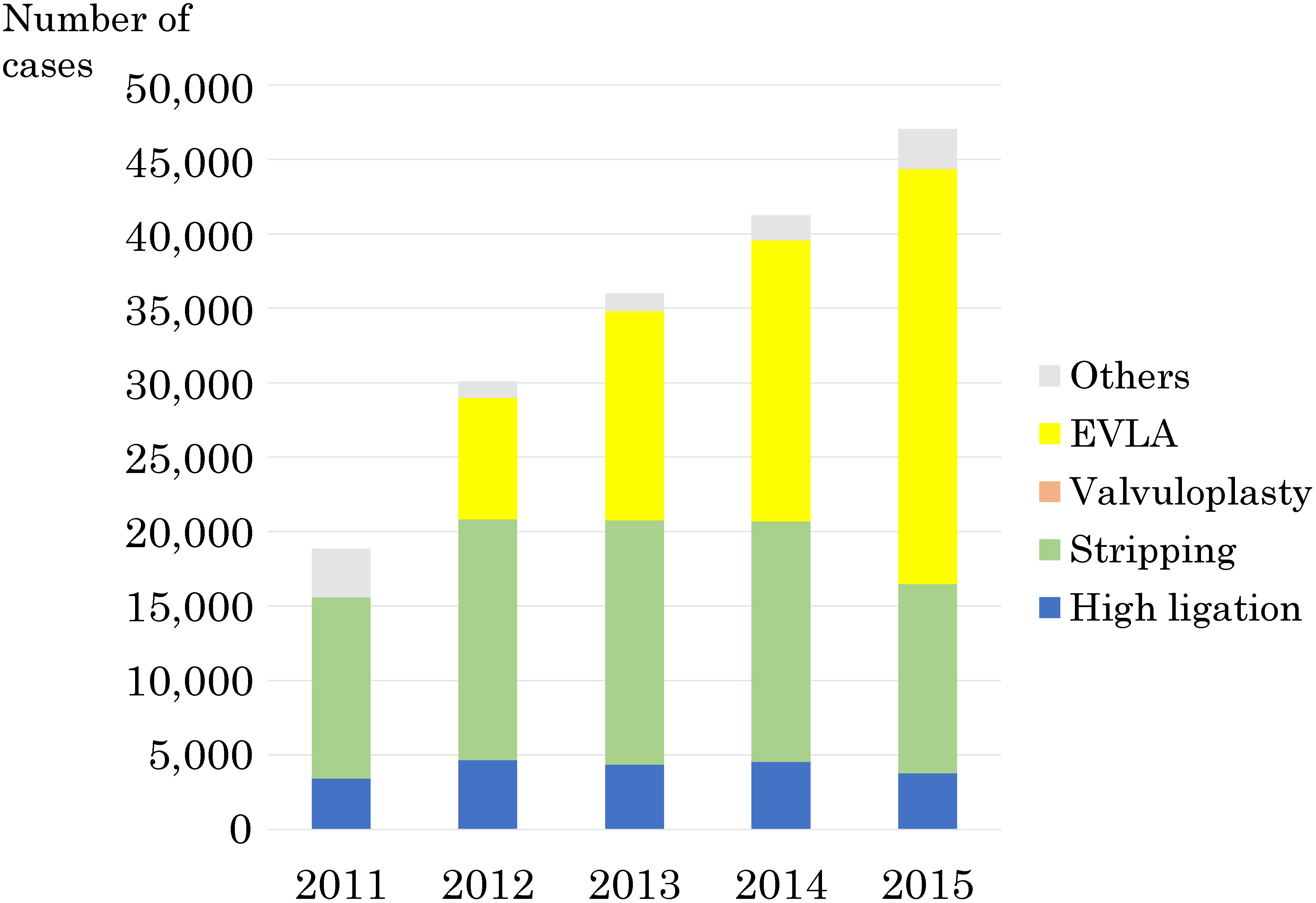
Fig. 6 Changes of varicose veins treatment in year 2011, 2012, 2013, 2014, and 2015.

The number of varicose vein operations markedly increased since 2011, reaching 47,046 cases in 2015, which was a 2.5-fold increase from 2011. By modality, the numbers of stripping (± sclerotherapy) operations and high ligation operations decreased, whereas the number of endovenous laser ablation (EVLA) (± sclerotherapy) increased to 27,849 (59.2%) cases (**Fig. 6**). This was probably due to the insurance coverage of 1470-nm laser devices that took effect in 2014 in Japan. Given that radiofrequency ablation apparatuses were also approved in 2014, endovascular cauterization techniques such as EVLA and radiofrequency ablation was considered to become the mainstream for treating varicose veins in the lower extremities.^8)^

**Table:** Table 7 Venous surgery Table 7-1 Varicose veins

Varicose veins treatment	Cases*^35)^	Male	Female	30-day mortality
High ligation±sclerotherapy	3,777	1,234	2,543	0
Stripping±sclerotherapy	12,715	5,084	7,631	1
Valvuloplasty	5	1	4	0
EVLA±sclerotherapy*^36)^	27,849	9,655	18,193	0
Others	2,700	815	1,885	0
Total	47,046	16,789	30,256	1

＊35) Only one procedure can be registered in one leg. ＊36) EVLA: endovenous laser ablation

EVLA: endovenous laser ablation

### 2) Deep vein thrombosis (including deep vein stenosis/obstruction) (Table 7-2)

Overall, 531 surgical cases were registered, of which 311 (58.6%) received inferior vena cava filter placement. Then, 135 (25.4%) cases had filter removal. Catheter-directed thrombolysis (CDT) was performed in 51 (9.6%) cases. Endovascular treatment for stenosis was performed in 11 (2.1%) cases, which were slightly increased from the previous year. By surgical modality, thrombectomy, bypass (peripheral vein revascularization), and venous stenosis removal (direct delivery) operations were performed in only 64 (12.1%), 6 (1.1%), and 2 (0.4%) cases, respectively.

**Table:** Table 7-2 Deep vein thrombosis (including venous stenosis or obstruction)

Deep vein thrombosis treatment	Cases	Male	Female	30-day mortality
Thrombectomy	64	34	30	0
Catheter-directed thrombolysis*^37)^	51	23	28	0
Bypass (peripheral venous reconstruction)	6	2	4	0
IVC filter insertion*^38)^	311	143	168	5
IVC filter retrieval*^38)^	135	62	73	1
Direct surgery of stenosis*^39)^	2	1	1	0
Endoluminal treatment of stenosis	32	10	22	0
Others	11	4	7	0
Total	531	240	291	6

＊37) Including the catheter-directed thrombolysis using hydrodynamic thrombectomy catheter. ＊38) Including temporary IVC filter. ＊39) Including obstruction.

### 3) Venous stenosis/obstruction in the upper extremities and the cervical region (Table 7-3)

The number of surgical operations was 115, which decreased from 2014. The most prevalent surgery was venous stenosis removal by endovascular treatment, which was performed in 67 (58.3%) cases.

**Table:** Table 7-3 Upper limb vein stenosis or obstruction

Treatment of vein stenosis (obstruction)	Cases	Male	Female	30-day mortality
Thrombectomy	22	10	12	0
Catheter-directed thrombolysis*^40)^	1	0	1	0
Bypass	12	8	4	0
SVC filter insertion*^41)^	1	0	1	0
Direct surgery of stenosis	2	2	0	0
Endoluminal treatment of stenosis	67	47	20	3
Others	15	9	6	0
Total	115	74	41	3

＊40) Including the catheter-directed thrombolysis using hydrodynamic thrombectomy catheter. ＊41) Including temporary IVC filter.

### 4) Vena cava reconstruction (Table 7-4)

The number of surgical operations was 75. Of these, 56 (74.7%) were inferior and 19 (25.3%) were superior vena cava/primary branch reconstructions. The most prevalent cause of illness was tumor, which occurred in 67 (89.3%) cases. There were 4 operative (5.3%) and 7 hospital (9.3%) deaths, which were fewer than those in 2014. By surgical modality, there were 16 cases of replacement, 9 cases of patch plasty, and 4 cases of bypass; the use of ePTFE was most prevalent.

**Table:** Table 7-4 Vena cava reconstruction

Vena cava reconstruction	Cases	Mortality		Etiology			Treatment procedures					Material for open surgery			
		30-day mortality	Hospital mortality	Tumor	Thrombus	Others	Patch plasty	Bypass	Replacement	PTA±stent	Others	Autogenous vessel	Polyester	ePTFE	Others
SVC reconstruction	19	1	2	16	2	1	2	4	7	5	1	1	4	8	2
IVC reconstruction	56	3	5	51	0	5	7	0	9	2	40	2	4	8	3
Total	75	4	7	67	2	6	9	4	16	7	41	3	8	16	5

Abbreviations; IVC: inferior vena cava, SVC: superior vena cava

### 5) Budd-Chiari syndrome (Table 7-5)

Percutaneous shunting was performed in only 2 cases, both of which were implemented in the Kyushu region.

**Table:** Table 7-5 Budd-Chiari syndrome

Treatment	Cases	Gender		Mortality		Material for open surgery			
		Male	Female	30-day mortality	Hospital mortality	Polyester	ePTFE	Autogenous vessel	Others
Shunting	0	0	0	0	0	0	0	0	0
Percutaneous shunting	2	1	1	0	0	0	1	1	0
Surgical recanalization	0	0	0	0	0	0	0	0	0
Total	2	1	1	0	0	0	1	1	0

### 6) Others (Table 7-6)

Deep venous aneurysm plication was performed in 64 cases in 2013, 25 cases in 2014, and 14 cases in 2015, showing year-on-year decreases.

**Table:** Table 7-6 Other surgery

Treatment	Cases	Gender		Mortality		Material for open surgery			
		Male	Female	30-day mortality	Hospital mortality	Polyester	ePTFE	Autogenous vessel	Others
Plication of deep venous aneurysm*^42)^	14	4	10	0	0	0	0	0	0
Plication of abdominal venous aneurysm	4	3	1	0	0	0	0	0	0
Others	1,050	598	452	35	68	0	0	0	0
Total	1,068	605	463	35	68	0	0	0	0

＊42) Including patch plasty.

## 7. Other Vascular Diseases and Related Surgical Operations (Table 8)

Compared with 2014, no significant changes were noted in 2015, other than the significant increase in the number of vascular access surgeries.

**Table:** Table 8 Other vascular diseases Table 8-1 Popliteal artery entrapment syndrome

Treatment	Cases	30-day mortality
Myotomy	12	0
Revascularization	31	2
Total	34	2

### 1) Popliteal artery entrapment syndrome and adventitial cyst (Tables 8-1, 8-2)

These are rare diseases to begin with; as such, no great changes were noted between 2014 and 2015.

**Table:** Table 8-2 Adventitial cystic disease

Treatment	Cases	30-day mortality
Cyst excision ± patch plastry	22	0
Replacement	13	0
Bypass	6	1
Total	36	1

### 2) Thoracic outlet syndrome (Table 8-3)

It continues to be a rare disease, with only 10 cases reported both in 2014 and 2015.

**Table:** Table 8-3 Thoracic outlet syndrome (TOS)

Treatment	Cases	Male	Female	30-day mortality	Type of TOS*^43)^		
					Neurogenic	Venous	Arterial
Rib resection*^44)^	1	1	0	0	1	0	0
Rib resection+scalenectomy	3	2	1	0	1	0	2
Bypass	7	5	2	0	0	4	3
Total	10	7	3	0	2	4	4

＊43) In the case with mixture type, the type having the most significant impact on the clinical symptom is listed. But, if the impacts are similar, multiple response is allowed. ＊44) Including cervical rib.

### 3) Vascular access surgery (Table 8-4)

The number of operations increased by 3,000 from the previous year, and it was expected to continue increasing as the dialysis population grows.

**Table:** Table 8-4 Vascular access operation

Treatment	Cases	30-day mortality
Arteriovenous access creation by autogenous material	13,511	100
Arteriovenous access creation by artificial material*^45)^	3,006	44
Open surgery for access repair	2,397	44
Endovascular access repair	8,200	34
Arterial transposition	477	15
Arteriovenous access aneurysm repair	469	3
Total	28,060	240

＊45) Including cases with access repair using artificial graft.

### 4) Lymphedema surgery (Table 8-5)

Compared with 2013, the number almost halved in 2014 but returned to the original level in 2015.

**Table:** Table 8-5 Surgery for lymphedema

Treatment	Cases	Male	Female	30-day mortality
Lymphovenous anastomosis	0	0	0	0
Lymph drainage operation	0	0	0	0
Resection	102	69	33	1
Total	102	69	33	1

### 5) Sympathectomy (Table 8-6)

The number of surgeries was 25 in 2015, which was almost unchanged from the levels in 2013 and 2014.

**Table:** Table 8-6 Sympathectomy

Sympathectomy	Cases	30-day mortality
Thoracic sympathectomy	18	0
Lumbar sympathectomy	7	0
Total	25	0

### 6) Upper/lower limb amputation (Tables 8-7, 8-8)

The number of upper limb amputations was unchanged in 2015, whereas that of lower limb amputations increased successively from the past 2 years. This suggests an increase in critical limb ischemia cases.

**Table:** Table 8-7 Amputation of upper limb

Amputation level	Cases	30-day mortality
Digit	19	1
Forearm / upper arm	4	1
Total	23	2

**Table:** Table 8-8 Amputation of lower limb*^46)^

Amputation level	Cases	30-day mortality	Etiology			
			ASO	DM-ASO	TAO	Others
Toe	615	14	240	311	4	60
Transmetatarsal	253	10	57	163	0	33
Lisfranc / Chopart	43	2	10	30	0	3
Syme	6	1	0	6	0	0
Below-Knee	265	12	92	150	1	22
Through-Knee / Above-Knee	324	22	154	122	1	47
Hip	5	0	2	0	0	3
Total	1,511	61	555	782	6	168

＊46) Amputations not due to ischemia are not included. Abbreviations; ASO: arteriosclerosis obliterans, DM-ASO: diabetic ASO, TAO: thromboangiitis obliterans (Buerger’s disease)

## Conclusion

Since 2011 when NCD registration was initiated, the overview of vascular surgery has been reported annually. This report reveals the overview in 2015 and gives the reader a glimpse of the present state of vascular surgery that has been changing over the years.

One major purpose of participating in the NCD is to improve the quality of medical services by the effective use of its data. Since data items need to be entered between busy work hours, entries should be limited to only the critical data items. However, the number of entry items has been increasing yearly from 2011 to 2015 to improve the evaluation of the quality of medical care. Fortunately, the operative mortality from vascular surgery (except surgical operations for aorta) was low; hence, this could not be used as an evaluation index. The future goal is to implement a new function in the NCD for comparing the quality of risk-adjusted vascular surgical treatment provided at our institution with the national standards. In 2018, the JSVS initiated a nationwide multicenter observational study on therapeutic options for laparotomy and stent-graft deployment for ruptured abdominal aortic aneurysm. The organization also started a retrospective study on infectious abdominal aortic aneurysm and common iliac artery aneurysm as a model study; in 2019, it started another retrospective study on the therapeutic modalities for popliteal artery entrapment syndrome and its prognosis. Through these studies, the JSVS has been attempting to solve various challenges. In addition, a clinical research promotion study was initiated in 2018, in which the modalities for synthetic vascular graft/stent-graft infection in the abdominal aortic region and its prognosis were investigated. For patients with arteriosclerosis obliterans and critical limb ischemia, the effects of malignant neoplasms on their prognosis were examined. For patients with ischemic limbs, the factors affecting the development of bypass wound complications were analyzed. In 2019, a multicenter observational study was initiated on cooperation between medical institutions engaged in emergency care for the aortic and peripheral arteries. The results of bypass surgery for patients with critical ischemia caused by collagen disease and angiitis in Japan were disclosed. In addition, in 2019, we started accepting novel research topics in the vascular surgical field from the public using the NCD data. To improve the reliability of the data, site visits also started in 2018.

In the future, the JSVS wishes to further develop the vascular surgery database on the NCD in collaboration with our dedicated members. We sincerely hope that this database will be of help to providing high-quality medical care for patients suffering from vascular diseases.

## Appendix

Team responsible for analyzing the 2015 annual report as follows;

Database Management Committee of the Japanese Society for Vascular Surgery: Nobuya Zempo (Chairman), Nobuyoshi Azuma (Vice-chairman), Yukio Obitsu (Vicechairman), Yoshinori Inoue, Jin Okazaki, Hideaki Obara, Hirono Satokawa, Kunihiro Shigematsu, Ikuo Sugimoto, Hiroshi Banno, Naoki Fujimura, Akihiro Hosaka, Shinsuke Mii, Noriyasu Morikage, Terutoshi Yamaoka, Tetsuro Miyata (Observer), Kimihiro Komori (Chief director of the Japanese Society for Vascular Surgery)

NCD Vascular Surgery Data analyzers: Arata Takahashi

## Additional Remarks

This annual report was published in the Japanese Journal of Vascular Surgery Vol. 29 (2020) No. 3; however, after publication errors in the numerical data and resulting errors in the table were detected, the corrections in the report were published in Vol. 30 (2021) No. 5 of the same journal. This English translation reflects those corrections.

## Notes

This is a translation of Jpn J Vasc Surg 2020; 29: 161–179.
